# Type Three Vasa Previa Diagnosed in Late Pregnancy Despite Apparent Placental Migration: A Case Report

**DOI:** 10.7759/cureus.104970

**Published:** 2026-03-10

**Authors:** Saki Aramaki, Toyofumi Hirakawa, Ayako Sanui, Daichi Urushiyama, Fusanori Yotsumoto

**Affiliations:** 1 Department of Obstetrics and Gynecology, Fukuoka University Hospital, Fukuoka, JPN

**Keywords:** placental migration, prenatal diagnosis, screening strategies, transvaginal sonography, type 3 vasa previa, type three vasa previa

## Abstract

Type three vasa previa is a rare and potentially fatal obstetric condition in which unprotected fetal blood vessels traverse the cervical membranes near the internal os despite normal placental morphology and umbilical cord insertion. Owing to an absence of classical risk factors, the prenatal diagnosis of vasa previa is particularly challenging; therefore, the condition may go undiagnosed, especially after apparent placental migration. A 36-year-old multiparous woman was referred to our clinic at 33 weeks and three days of gestation with suspicions of a low-lying placenta. Follow-up sonography performed at 35 weeks and two days of gestation revealed an apparent placental migration to the normal position; however, a detailed transvaginal color Doppler sonogram incidentally revealed a pulsatile fetal blood vessel crossing the internal os. The patient was diagnosed with type three vasa previa and underwent an elective cesarean delivery at 35 weeks and three days of gestation, resulting in the birth of a healthy male infant. A postnatal examination of the placenta and umbilical cord confirmed the presence of a membrane-traversing fetal blood vessel in the absence of a velamentous cord insertion or accessory placental lobes. This case highlights a critical diagnostic pitfall, and the apparent resolution of placental malposition does not exclude the presence of type three vasa previa. Therefore, continued third-trimester surveillance with transvaginal color Doppler sonography is essential to avoid missed diagnoses and prevent a potentially catastrophic fetal hemorrhage.

## Introduction

Vasa previa is a rare but potentially life-threatening obstetric condition in which fetal blood vessels traverse the cervical membranes near the internal os (the opening of the cervical canal into the uterine cavity), unprotected by Wharton’s jelly (the protective gelatinous connective tissue surrounding umbilical vessels) or placental tissue. If the condition remains undiagnosed and the cervical membranes rupture, the fetal blood vessels may tear, resulting in rapid exsanguination and death. Perinatal mortality exceeds 50% in undiagnosed cases, decreasing to less than 10% when the condition is identified prenatally; therefore, the antenatal diagnosis of vasa previa is crucial for ensuring the best fetal outcomes [1,2]. Vasa previa has traditionally been classified into two types: type one, which is associated with a velamentous cord insertion, in which umbilical vessels traverse the membranes without protection by Wharton’s jelly, and type two, which is associated with accessory or bilobed placentation. A third variant of vasa previa, type three, was recently proposed, characterized by aberrant fetal vessels traversing the cervical membranes near the internal os despite a normal umbilical cord insertion and the absence of placental abnormalities [3,4]. Although the overall incidence of vasa previa is approximately one in 2,500-6,000 pregnancies [5], the incidence of type three remains unknown owing to its rarity and diagnostic difficulty. In contrast to vasa previa types one and two, type three lacks consistently identifiable structural abnormalities such as velamentous cord insertion or bilobed placenta. However, its risk profile overlaps with that of classic vasa previa, as assisted reproductive technology (ART) and low-lying placenta have also been reported. Because these risk factors are nonspecific, prenatal suspicion and diagnosis remain challenging [6-8]. Although transvaginal sonography combined with color Doppler imaging remains the most effective screening modality for vasa previa, some type three cases may be overlooked when the placental morphology appears normal, placental position improves, or no risk factors are present [9,10]. We report herein a case of type three vasa previa diagnosed in the late third trimester after the apparent resolution of a previously identified low-lying placenta. This case highlights an important diagnostic pitfall associated with vasa previa and the necessity of continued third trimester monitoring with transvaginal color Doppler sonography, even after the placenta migrates to a seemingly normal position.

## Case presentation

**Figure 1:**
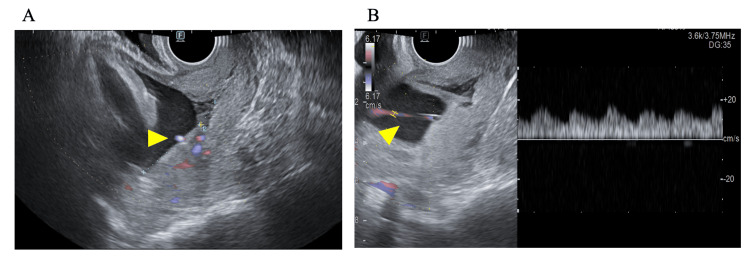
Transvaginal ultrasonography (A) A thin fetal blood vessel (yellow arrowhead) is seen traversing the cervical membranes directly over the internal os, suggestive of vasa previa, although no placental abnormalities are observed; (B) Color Doppler sonography demonstrates arterial waveforms consistent with the umbilical artery within the aberrant blood vessel.

A 36-year-old woman (gravida seven, para two: two spontaneous vaginal deliveries, two spontaneous abortions, and two induced abortions) with no notable medical or family history conceived spontaneously and was undergoing routine antenatal care at an obstetric clinic. Fetal growth was appropriate for the assumed gestational age, and all maternal laboratory values were within normal limits. A transvaginal ultrasound performed at the referring clinic at 31 weeks of gestation raised the suspicion of an abnormal placental location. Upon referral to our clinic at 33 weeks of gestation, sonography revealed posterior placental implantation with the placental edge located 11 mm from the internal os, consistent with a diagnosis of low-lying placenta, although no abnormal umbilical cord vessels were observed. Given the prior diagnosis of a low-lying placenta, a targeted transvaginal examination with color Doppler was performed at 35 weeks and two days of gestation as part of the follow-up evaluation. At 35 weeks and two days of gestation, follow-up transvaginal sonography demonstrated the migration of the placental edge to 37 mm from the internal os, indicating a normalization of the placental position. However, color Doppler imaging obtained at the same time revealed a fetal vessel traversing the cervical membranes near the internal os with an arterial blood flow pattern (Figure 1).

**Figure 2:**
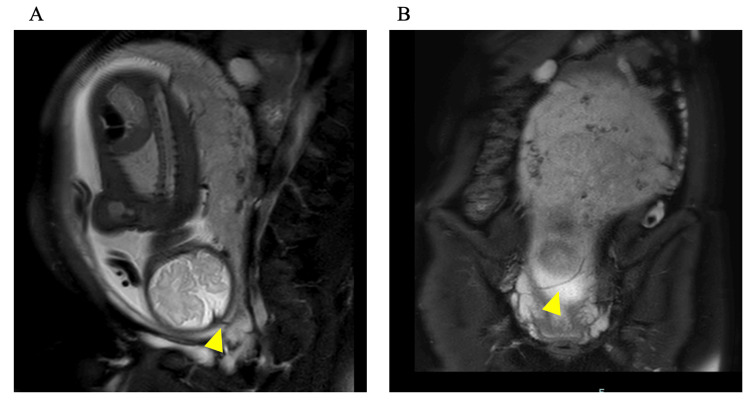
Pelvic magnetic resonance imaging (MRI) (A) Sagittal and (B) coronal views. The yellow arrowheads indicate a fetal vessel running within the cervical membranes near the internal os. The vessel is seen coursing along the posterior uterine wall, consistent with vasa previa.

Subsequent magnetic resonance imaging (MRI) of the pelvis confirmed the presence of a fetal vessel running along the cervical membranes adjacent to the internal os, resulting in a definitive diagnosis of vasa previa (Figure 2).

**Figure 3:**
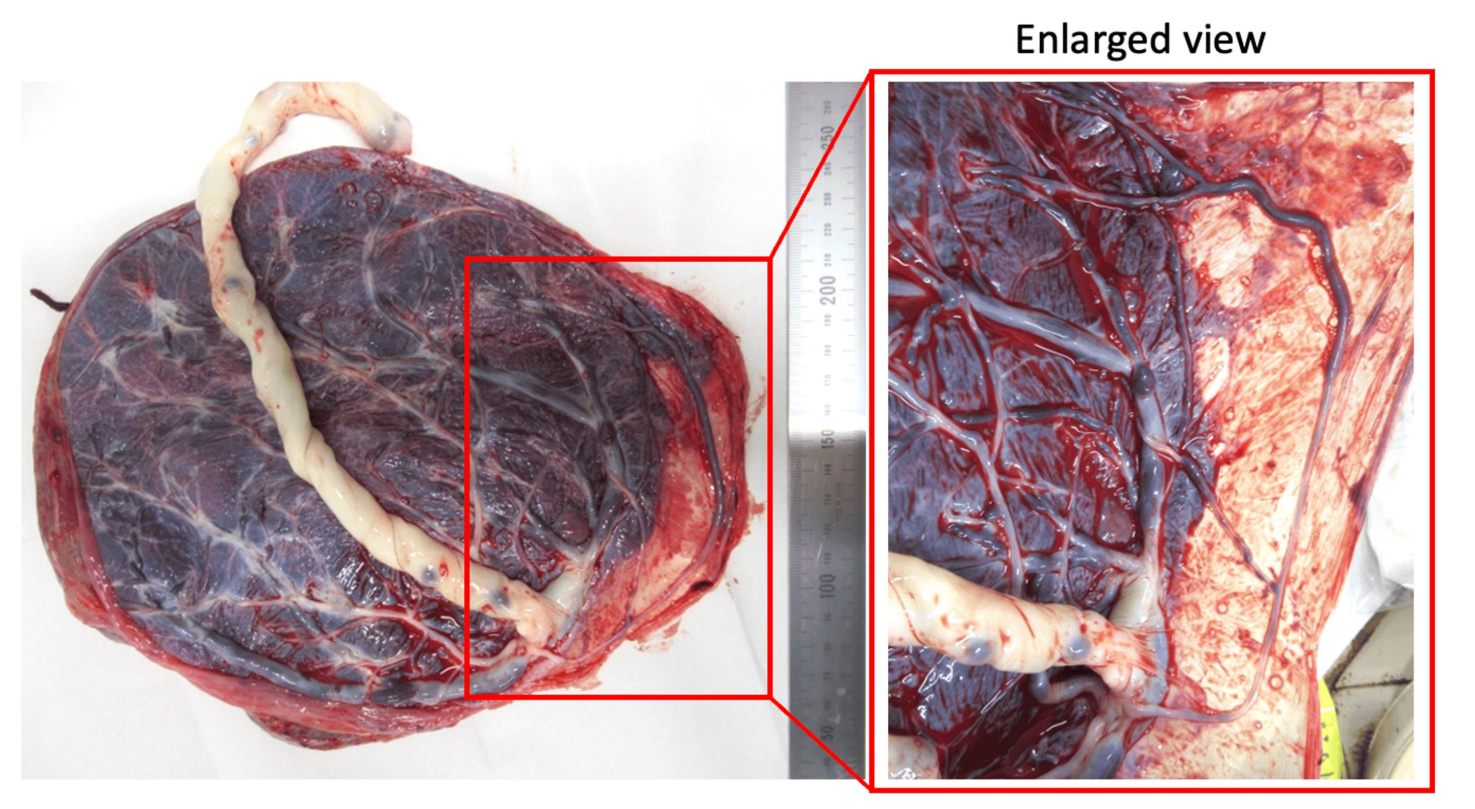
Gross image of the placenta and umbilical cord after delivery The umbilical cord is marginally inserted into the placental edge. An unprotected fetal vessel was visible traversing the membrane, with an estimated length of approximately 14 cm. No accessory placenta was observed.

The patient was admitted as an inpatient the same day and subsequently underwent a cesarean section at 35 weeks three days of gestation. A male neonate weighing 2,782 g and measuring 48 cm in length was delivered, with Apgar scores of seven at 1 min and 5 min. Umbilical arterial blood gas analysis revealed a pH of 7.33. The patient’s postoperative course was uneventful, and she was discharged on postoperative day five. The neonate was admitted to the neonatal intensive care unit on the day of delivery due to prematurity, received treatment for transient tachypnea and neonatal hypoglycemia, and was discharged at 15 days of age. A macroscopic examination of the placenta revealed no lobulations or parenchymal cord insertions; however, a single umbilical artery traversing approximately 14 cm along the membranes was identified, confirming the diagnosis of type three vasa previa (Figure 3).

## Discussion

The present case demonstrates the diagnostic challenges inherent with type three vasa previa, a rare variant that lacks the classical risk factors associated with types one and two. In this case, the low-lying placenta identified at 33 weeks of gestation migrated to a normal position by the next ultrasound, leading to a reduced index of suspicion. Detailed transvaginal color Doppler sonography obtained at 35 weeks and two days of gestation revealed a pulsatile fetal vessel crossing the internal os, resulting in a timely diagnosis of vasa previa type three followed by a planned cesarean delivery with a favorable neonatal outcome.

Contrary to vasa previa types one and two, which are typically suspected based on identifiable risk factors such as a velamentous cord insertion or accessory placental lobes, type three occurs in the absence of such findings. Its defining feature is an aberrant fetal vessel traversing the cervical membranes over the internal os despite a normal appearing placenta and cord anatomy [3-5]. In the present case, the apparent resolution of the low-lying placenta further reduced clinical suspicion of vasa previa, illustrating a critical diagnostic pitfall of this condition. Although the diagnosis in this case was established based on transvaginal sonography with color Doppler findings, fetal MRI was performed as an adjunct to further delineate the anatomical relationship between the aberrant vessel and the internal os and to exclude velamentous cord insertion or the presence of an accessory placenta. MRI is not routinely required for the diagnosis of vasa previa when transvaginal Doppler findings are conclusive; however, it may be reserved for selected cases with inconclusive sonographic findings or complex anatomy requiring additional clarification. Although MRI is not routinely warranted, it may serve as a useful adjunct when anatomical clarification is needed. However, the embryological basis of type three vasa previa remains unclear. It has been hypothesized that the aberrant development of fetal blood vessels during placental implantation or vascular remodeling associated with placental migration and/or marginal cord insertion may lead to the traction and subsequent displacement of these vessels toward the cervical os [2,9-11]. Although several hypotheses have been proposed, the embryological basis of type three vasa previa remains poorly understood. It has been suggested that aberrant vascular development during placental implantation or remodeling associated with placental migration and marginal cord insertion may contribute to the displacement of fetal vessels toward the cervical os. However, clear mechanistic evidence is lacking, and these explanations remain theoretical. Further research is required to elucidate the precise developmental pathways underlying this condition.

To contextualize our findings around this case, we reviewed five previously published reports on type three vasa previa (Table 1) [5,11-14].

**Table 1:** Comparison of reported cases of type three vasa previa The gestational age at diagnosis, imaging modality, cord insertion site, vessel length, and unique features are summarized for each case. GA, gestational age; TV-US, transvaginal ultrasound; MRI, magnetic resonance imaging; ART, assisted reproductive technology.

	Diagnosis GA (weeks)	Delivery GA (weeks)	Trigger for detection	Imaging modality used	Cord insertion
Mohamad et al. [5]	38	38	Intrapartum (during induction of labor)	TV-US	Marginal
Oyelese [11]	32	NR	Follow-up after resolution of placenta previa	TV-US	NR
Kim et al. [12]	35	36	Follow-up after resolution of low-lying/previa	TV-US	Normal
Shiro et al. [13]	26	34	Asymptomatic prenatal screening	TV-US + MRI	Normal
Suekane et al. [14]	NR	NR	Asymptomatic prenatal screening	TV-US	Normal
Present case	35	35	Follow-up after resolution of low-lying placenta	TV-US + MRI	Marginal

A recent systematic review has summarized a larger number of reported cases; however, the aim of the present comparison was to perform detailed case-level analysis using uniformly reported variables. Therefore, we focused on individual case reports that allowed direct comparison with our case. The reported gestational ages at diagnosis ranged from 26 to 38 weeks, with several cases identified during third trimester follow-up examinations after an apparent improvement in placental position. Importantly, apparent migration of the placental edge does not necessarily indicate resolution of vasa previa, as unprotected fetal vessels may persist over the internal os despite changes in placental location. The present case, detected at 35 weeks and two days of gestation, after the documented placental migration, represents one of the later antenatal diagnoses of type three vasa previa reported to date. Consistent with the previous cases, there were no accessory placental lobes, velamentous cord insertions, or ART-related risk factors. Of note, the exposed fetal blood vessel in this case measured approximately 14 cm in length, a detail not often described in previous reports but potentially useful for anatomical recognition. Among the reported cases, transvaginal sonography with color Doppler was the principal diagnostic modality, with MRI used adjunctively in only a few of the cases, including ours [15,16]. Previously reported case series indicate that type three vasa previa is most commonly detected during late second- or third-trimester follow-up examinations, often after apparent improvement in placental position. Our case is consistent with these observations, further illustrating that risk-based follow-up imaging plays a central role in detection and that favorable outcomes are achievable when diagnosis is established before labor. Collectively, these observations reinforce the message that the apparent normalization of placental position does not preclude the presence of vasa previa.

Accordingly, third trimester follow-ups should include a detailed transvaginal color Doppler evaluation of the internal os, particularly in patients with a history of placenta previa or low-lying placenta [17,18]. In the present case, placental cord insertion was systematically evaluated during the mid-trimester anatomy scan, and no abnormal findings were identified at that time. Despite adherence to recommended screening practices, type three vasa previa was not suspected until the third trimester. This observation highlights a limitation of current antenatal screening strategies, as type three vasa previa may remain undetectable despite apparently normal mid-trimester findings. Although screening strategies vary, from routine documentation of cord insertion at mid-pregnancy scans to targeted late pregnancy reassessments, there is a growing consensus that structured protocols improve the prenatal detection of vasa previa and facilitate appropriate delivery planning [19,20]. Previous clinical series have reported high prenatal detection rates when careful transvaginal color Doppler imaging protocols are applied, with most cases diagnosed before labor and associated with favorable neonatal outcomes [21]. Antenatal diagnosis remains the key determinant of fetal outcomes in these cases, as perinatal prognosis is markedly improved when vasa previa is identified before labor [22]. As this report is limited by its single-case nature, further accumulation of similar cases is required to refine the screening strategies for type three vasa previa.

## Conclusions

The present case highlights that type three vasa previa can persist or become clinically apparent even after the placenta migrates to a seemingly normal position. Given the high prenatal detection rates and favorable outcomes associated with antenatal diagnosis, continued third-trimester surveillance using transvaginal color Doppler ultrasonography is warranted to avoid missed diagnoses and enable timely planned cesarean delivery. These recommendations are supported by cumulative evidence from previously reported studies, with the present case providing additional clinical corroboration. Apparent placental migration and a normal placental or umbilical cord presentation should never preclude continued sonographic surveillance for vasa previa.
